# Households experiencing catastrophic costs due to tuberculosis in Uganda: magnitude and cost drivers

**DOI:** 10.1186/s12889-020-09524-5

**Published:** 2020-09-16

**Authors:** Winters Muttamba, Racheal Tumwebaze, Levicatus Mugenyi, Charles Batte, Rogers Sekibira, Abel Nkolo, Achilles Katamba, Simon Kasasa, Robert Kaos Majwala, Stavia Turyahabwe, Frank Mugabe, Kaggwa Mugagga, Peter Lochoro, Seyoum Dejene, Estella Birabwa, Claudio Marra, Ines Garcia Baena, Bruce Kirenga

**Affiliations:** 1 Makerere University Lung Institute, College of Health Sciences, Makerere University, Kampala, Uganda; 2 University Research Co, LLC (URC) & Center for Human Services (CHS), Kampala, Uganda; 3 School of Medicine, College of Health Sciences, Makerere University, Kampala, Uganda; 4 School of Public Health, College of Health Sciences, Makerere University, Kampala, Uganda; 5 National Tuberculosis and Leprosy Program, Ministry of Health, Kampala, Uganda; 6 World Health Organization, Kampala, Uganda; 7 Doctors with Africa, CUAMM, Padua, Italy; 8 USAID Mission, Kampala, Uganda; 9 World Health Organization, Geneva, Switzerland

**Keywords:** Catastrophic costs, Dissaving, Direct medical costs, Indirect non-medical costs

## Abstract

**Background:**

Tuberculosis (TB) patients in Uganda incur large costs related to the illness, and while seeking and receiving health care. Such costs create access and adherence barriers which affect health outcomes and increase transmission of disease. The study ascertained the proportion of Ugandan TB affected households incurring catastrophic costs and the main cost drivers.

**Methods:**

A cross-sectional survey with retrospective data collection and projections was conducted in 2017. A total of 1178 drug resistant (DR) TB (44) and drug sensitive (DS) TB patients (1134), 2 weeks into intensive or continuation phase of treatment were consecutively enrolled across 67 randomly selected TB treatment facilities.

**Results:**

Of the 1178 respondents, 62.7% were male, 44.7% were aged 15–34 years and 55.5% were HIV positive. For each TB episode, patients on average incurred costs of USD 396 for a DS-TB episode and USD 3722 for a Multi drug resistant tuberculosis (MDR TB) episode. Up to 48.5% of households borrowed, used savings or sold assets to defray these costs. More than half (53.1%) of TB affected households experienced TB-related costs above 20% of their annual household expenditure, with the main cost drivers being non-medical expenditure such as travel, nutritional supplements and food.

**Conclusion:**

Despite free health care in public health facilities, over half of Ugandan TB affected households experience catastrophic costs. Roll out of social protection interventions like TB assistance programs, insurance schemes, and enforcement of legislation related to social protection through multi-sectoral action plans with central NTP involvement would palliate these costs.

## Background

Uganda is a high Tuberculosis/Human Immunodeficiency Virus (TB/HIV) burden country, and the Tuberculosis prevalence survey conducted in 2014 put the prevalence at 253 per 100,000 population [1] while data available for 2018 puts the incidence at 200 per 100,000 population [2]. The TB incidence among HIV positive individuals is 80 per 100,000 population while the mortality among the HIV co-infected is 32/100,000 population [3]. The proportion of multi drug resistant TB (MDR TB) among the new TB cases and previously treated TB cases is 1.6 and 12% respectively [3]. In 2018, Uganda notified 52,458 TB patients and 65% of these were male [4].

TB patients often navigate complex healthcare systems before and after a TB diagnosis has been made. This often results in them incurring large costs related to illness and disability, as well as seeking and receiving health care. Low income countries like Uganda have TB patients that face costs that could amount to half their annual income [5] despite TB services being provided free of charge in public health facilities [6]. In the private health facilities, patients incur costs of screening and diagnosis. For the private health facilities designated as diagnostic and treatment units (DTU), the TB drugs are provided free of charge. TB affects the poorest segment of society disproportionately and the poverty-aggravating effects of TB are therefore gravest for those who are already vulnerable [7].

To cushion TB patients against the costs, the Global TB Programme suggests several cross-sectoral measures including increasing insurance coverage, reimbursements, regulating and eliminating user fees, inclusion of TB patients in social protection schemes among others [7]. The end TB strategy has as one of the targets that no TB-affected household should face catastrophic costs due to tuberculosis care [8]. Catastrophic costs in most surveys have been set at 20% of the household’s annual income as this threshold is mostly associated with adverse TB outcomes [9].

While some countries may attempt to provide free services for TB related care, often only diagnostics and anti TB drugs are free and patients may face other TB-related expenses. Such include direct payments on transport, symptom relieving medications, food and indirect expenses due to lost income [9]. In Uganda, social protection services (cash transfers, food support, social insurance, housing, social assistance) are limited, with the MDR TB patients being prioritized. An unpublished report from one of the USAID funded projects (Strengthening Uganda’s Systems for Treating AIDS Nationally- SUSTAIN) indicates that MDR TB patients at the hospitals they support receive a refund of United States Dollars (USD) 1.4 every time they come to the health facility or a monthly lump sum of USD 32.0. The median monthly wage for people in paid employment is equivalent to USD 20.0 in rural areas and USD 57.0 in urban areas [10].

This survey was designed to ascertain the proportion of TB affected households experiencing catastrophic costs and to identify cost drivers in order to guide policies on cost mitigation and delivery model improvements. It measures the proportion of TB patients (and their households) that experienced catastrophic total cost in 2017.

## Methods

This survey followed World Health Organization (WHO) methodology and protocol design [7]. It was designed as a cross-sectional survey design with retrospective data collection and projections. The survey was conducted across TB diagnostic and treatment units (DTU) which report to the national TB program and were sampled through a cluster sampling strategy. A sample size of 1174 patients was selected from 67 of the 1680 DTUs. Patients were consecutively enrolled as they visited the health facility. Consecutive enrollment was continued till the number of patients allocated to the DTU was reached. In cases of children, the guardian accompanying them was interviewed and guardian costs calculated. The guardian costs (direct non-medical and direct medical) were included in the calculation of costs if the guardian was part of the same household of the patient. Clusters were allocated to 13 regions proportionately according to the TB notification rates.

All consecutive Drug Sensitive TB (DS-TB) and multi drug resistant TB (MDR-TB) patients registered for treatment who were attending a sampled facility for a follow-up visit (after a minimum of 2 weeks into the present intensive or continuation treatment phase) were interviewed using a questionnaire developed by WHO [11], and reported on expenditures, time loss, measures ability to pay (including assets ownership, household expenditures and income) and coping mechanisms (taking loans, selling assets, taking children out of school) retrospectively. Patients in each of the two treatment phases were interviewed at different time points during their treatment phase. Data collection for patients in different treatment phases allowed for the imputation of data and model projections of future and past costs during the entire illness episode.

### Costs of TB and MDR episodes

For each TB-affected household, total costs were calculated as the sum of direct medical costs, direct non-medical costs (transportation, accommodation, food, nutritional supplements) and indirect costs after the onset of TB symptoms and while in care as per WHO definitions [7]. Costs for food and nutritional supplements included food required during hospitalization or food and nutritional supplements recommended and additional to the regular food basket. Patients were asked if they have had to buy any additional food e.g. meat, fruits, energy drinks, or nutritional supplements e.g. multivitamins outside their regular diet because of TB as recommended by the health care staff.

Indirect costs were calculated using reported time used while seeking and receiving care during the TB episode (in hours) multiplied by an individual hourly rate derived from self-reported hourly income which was calculated based on the reported individual income in conjunction with the reported hours worked (so-called the human capital approach) [7], assuming that hours lost would have been used for a productive activity. Annual household expenditures were calculated as the sum of weekly, monthly and annual reported expenditures. The household expenditure questions excluded consumption that is not based on market transactions and included validated questions from a household consumption survey questionnaire.

### Catastrophic cost calculation

To ascertain the proportion experiencing catastrophic costs, our main analysis used the human capital approach paired with household expenditures as a measure of ability to pay for health. Household expenditures were the money payments or the incurrence of liability to obtain goods and services. While we collected assets and reported income, household expenditures appeared more robust as this could easily be collected at the facility. Catastrophic costs were calculated as total costs (indirect and direct combined) exceeding 20% of the household’s annual expenditure.

In addition to catastrophic cost calculations, data collected allowed for assessment of dissaving strategies, evaluation of risk factors for incurring catastrophic costs and calculation of the proportion of TB-affected households below the poverty line (i.e. living on less than USD 1.9 per day) before and after contracting the disease (impoverishment).

Impoverishment was calculated as the proportion of households with daily expenditure below 6760 Uganda Shillings (2017) which is equivalent to 1.90 US$ (2011 international poverty line). The proportion below poverty (before TB) was calculated as the number with monthly individual income (pre-TB) below the monthly poverty threshold.

To obtain those pushed below poverty due to TB, we added total costs (from output approach) to individual income pre-TB, and checked the number falling below the threshold.

Similarly, for those pushed below poverty level due to direct medical and non-medical costs we added these costs in and recalculated the proportion below threshold.

### Data collection process and analysis

This facility-based survey collected data at 67 health facilities across the country. Data were collected electronically by trained research assistants using a mobile and web -based system (ONA, https://ona.io/home/) downloaded onto tablets, collected off-line and uploaded when online. Part of the data collected was from TB cards and registers while the rest were collected by interviewing eligible patients at the facility for around 1 hour.

Data cleaning and analysis was done in Stata® Version 13 (StataCorp. 2013) in line with WHO minimum reporting formats [7].

Results were adjusted for survey design and presented by household expenditure quintiles where appropriate (e.g. dissaving strategy).

## Results

Table 1 shows the socio-demographic and clinical characteristics of the respondents. The DS-TB respondents were 1134 (96.2%) while the MDR-TB respondents were 44 (3.7%). Males, 739 (62.7%) were more than women, 439 (37.3%). Up to 362 (30.8%) respondents were in the age group of 25–34 years and this accounted for the highest number of respondents. The HIV positive respondents in this survey were 654 (55.5%) while the respondents that had previously been treated for TB were 103 (8.7%), with the proportion higher among the MDR-TB patients; 28 (64%) than DS-TB patients; 75 (6.6%). Up to 618 (52.5%) patients were interviewed while they were in the continuation phase of TB treatment. Under a half (48.3%) of the respondents had attained primary school education.

**Table 1:** Socio-demographic and clinical characteristics of the respondents (unweighted)

	MDR-TB		DS-TB		Overall	
	Sample (weighted)	National	Sample (weighted)	National	Sample (weighted)	National
N	**44**	**1100**	**1134**	**43,413**	**1178**	**45,284**
**Socio-demographic characteristics of survey sample**						
*Sex, N (%)*						
Male	30 (67.9%)		709 (62.5%)		739 (62.7%)	73%
Female	14 (32.1%)		425 (37.5%)		439 (37.3%)	28%
*Age (%)*						
0–14	2 (5.1%)		54 (4.8%)		57 (4.8%)	10%
15–24	5 (11.3%)		159 (14%)		164 (13.9%)	90%
25–34	14 (31.3%)		349 (30.7%)		362 (30.8%)	
35–44	11 (24.4%)		294 (25.9%)		304 (25.8%)	
45–54	9 (21.5%)		159 (14.1%)		169 (14.3%)	
55–64	0 (0%)		74 (6.5%)		74 (6.3%)	
65+	3 (6.6%)		45 (4%)		48 (4.1%)	
*Patient’s (guardian’s) education status %*						
Not yet started school	8 (18.8%)		151 (13.3%)		159 (13.5%)	
Primary school	23 (53%)		546 (48.2%)		570 (48.3%)	
Secondary school	12 (26.5%)		315 (27.8%)		327 (27.7%)	
Tech/Tertiary School	0 (0%)		75 (6.6%)		75 (6.4%)	
University and higher	1 (1.7%)		46 (4.1%)		47 (4%)	
*Occupation pre-disease*						
Professionals	2 (5.3%)		70 (6.2%)		72 (6.1%)	
Technicians and associate professionals	0 (0%)		33 (2.9%)		33 (2.8%)	
Clerical support workers	1 (2.8%)		7 (0.6%)		8 (0.7%)	
Service and sales workers	15 (34.2%)		272 (24%)		287 (24.4%)	
Skilled agricultural, forestry and fishery workers	0 (0%)		21 (1.8%)		21 (1.8%)	
Craft and related trades workers	2 (4.3%)		56 (4.9%)		58 (4.9%)	
Plant and machine operators, and assemblers	0 (0%)		6 (0.6%)		6 (0.5%)	
Elementary occupations	4 (10.3%)		225 (19.8%)		229 (19.4%)	
Armed forces	2 (3.6%)		14 (1.2%)		15 (1.3%)	
Other	2 (3.9%)		76 (6.7%)		78 (6.6%)	
**Clinical Characteristics**						
*Phase, N (%)*						
Intensive	18 (41.9%)		541 (47.7%)		560 (47.5%)	
Continuation	25 (58.1%)		593 (52.3%)		618 (52.5%)	
*Recorded HIV Status, N (%)*						
Positive	25 (57.3%)		487 (42.9%)		654 (55.5%)	40%
Negative	19 (42.7%)		636 (56%)		512 (43.4%)	53%
Unknown	0 (0%)		12 (1.1%)		12 (1%)	7%
*Retreatment status, N (%)*						
New	16 (36%)		1060 (93.4%)		1075 (91.3%)	
Retreatment/Relapse	28 (64%)		75 (6.6%)		103 (8.7%)	

Table 2 below highlights the model of care patients were receiving at the time of interview i.e. whether they were ambulatory or hospitalized. More MDR-TB patients than DS-TB patients were hospitalized i.e. 18 (41.9%) vs 74 (6.5%). The MDR-TB patients were hospitalized more times than the DS-TB patients (2 vs 1) and on average, the MDR-TB patients were hospitalized for 91 days while the DS-TB patients were hospitalized for 13 days.

**Table 2:** Model of care

	MDR-TB	DS-TB
	44	1134
	Mean (95% CI)	Mean (95% CI)
**Hospitalisation**		
Hospitalized at time of interview, N (%)	18 (41.9%)	74 (6.53%)
Previously hospitalized during current phase, N (%)	7 (16.7%)	125 (11.0%)
Times hospitalized during current phase, Mean (95% CI)	1.64 (0.83–2.45)	1.14 (1.04–1.23)
Mean duration (days) hospitalized during current phase (95% CI)	91.4 (0–199.2)	12.9 (10.1–15.8)
Median duration (days) hospitalized during current phase (IQR)	30 (26–102)	7 (5–14)
**Ambulatory care**		
Number of visits per episode: total (95% CI)	1093.4 (917–1269.8)	51.2 (42.1–60.3)
Number of visits: DOT (95% CI)	614.5 (555.6–673.5)	167.6 (157.7–177.5)
Number of visits: follow-up (95% CI)	10.9 (0–22.5)	3.7 (3.1–4.3)
Number of visits: drug pick-up (95% CI)	569.1 (529.9–608.3)	9.1 (7.7–10.5)
Number of visits pre-diagnosis (95% CI)	1.6 (0.9–2.2)	1.1 (1.1–1.2)
Proportion of first visits to primary health facilities	5 (84.2%)	189 (39%)
Proportion of first visits from private facilities	2 (28.8%)	159 (32.8%)
Proportion of TB diagnoses made at private or NGO facility	2 (5%)	300 (26.5%)
**Treatment duration**		
Treatment duration: intensive phase, weeks Mean (95% CI)	7 (6.1–7.9)	2 (2–2.1)
Treatment duration: continuation phase, weeks Mean (95% CI)	14.8 (12.8–16.8)	4.1 (4.1–4.1)
**Treatment delay (among new patients in intensive phase)**	**6**	**486**
Weeks of treatment delay Mean (95% CI)	9.5 (3.4–15.7)	9.9 (8.1–11.8)
Proportion of patients with delay > 28 days (%)	3 (50.0%)	223 (45.9%)

For ambulatory care and per TB episode, MDR-TB patients had more visits to the facilities than the DS-TB patients (1093 vs 51). The number of directly observed therapy (DOT) visits was 614.5 for the MDR-TB patients compared to 167.6 for the DR-TB patients, with more follow-up visits for the MDR-TB patients than the DR-TB patients (10.9 vs 3.7).

Among the MDR-TB patients, treatment was delayed by 9.5 weeks compared to 9.9 weeks among the DS-TB patients with 3 (50.0%%) of the MDR-TB patients and 223 (45.9%) of DS-TB patients delaying treatment by 28 days.

**Fig. 1:**
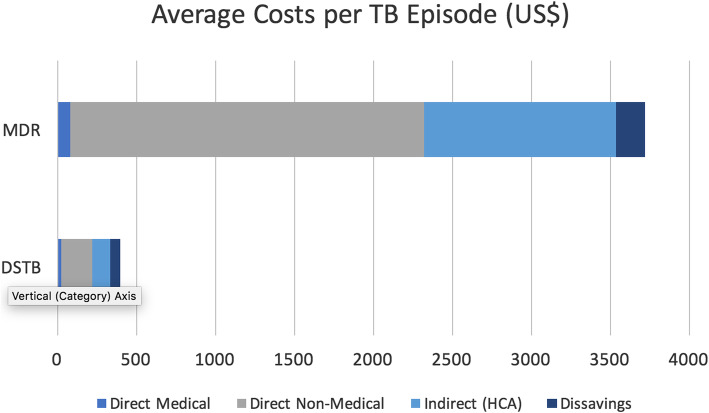
Average costs per TB episode

Table 3 summarizes the costs the patients and their guardians incurred both pre-diagnosis and post-diagnosis. Pre-diagnosis, the biggest drivers of costs were medical and travel for both MDR-TB and DS-TB. The biggest drivers of costs after a TB diagnosis was made were nutritional supplements (MDR-TB = US$ 1262, DS-TB = US$ 189) followed by travel (MDR-TB = US$ 1019, DS-TB = US$ 44) and food (MDR-TB = US$ 498, DS-TB = US$ 31). The non-medical costs were the biggest contributor of the costs for both types of TB. On average, it costs an MDR-TB patient US$ 3722 for an entire episode of TB while for DS-TB patients it costs US$ 396 for an entire TB episode. Figure 1 highlights that the biggest costs for both types of TB are direct non-medical followed by the indirect costs and direct medical costs.

**Table 3:** Estimated total costs borne by patients’ households affected by TB, MDR-TB or all, median breakdown (USD† 2017 (95% CI)

	Costs	MDR-TB Mean,95%CI	DS-TB Mean,95%CI	Overall Mean,95%CI
Pre-diagnosis	Medical	4.11(0.27–7.94)	8.55(2.80–14.31)	8.50(2.79–14.20)
	Travel	6.48(3.84–9.13)	2.10(1.39–2.81)	2.15(1.45–2.86)
	Accommodation	0(0–0)	0.34(0.10–0.58)	0.34(0.10–0.57)
	Food	0.69(0.41–0.97)	1.10(0.33–1.87)	1.09(0.33–1.85)
	Nutritional supplements	0.44(0.15–0.73)	1.08(0.32–1.84)	0.80(0.20–1.41)
	Hours lost by patient and guardian multiplied by hourly wage	1.52(0.97–2.08)	1.7(0.67–2.72)	1.69(0.67–2.71)
Post-diagnosis	Medical	78.7(12.5–145.0)	16.2(9.2–23.2)	18.5(11.2–25.9)
	Travel	1019(896–1143)	43.9(34.0–53.7)	79.9(51.0–108.8)
	Accommodation	0.4(0–1.1)	1.4(0–3.4)	1.4(0–3.3)
	Food	498(353–642)	30.6(15.8–45.5)	47.9(25.8–70.1)
	Nutritional supplements	1263.(928–1597)	189(151–227)	225(173–277)
	Caregiver (guardian) costs	115(0–248)	25.2(14.7–35.7)	27.9 (17.3–38.5)
	Hours lost by patient and guardian x Hourly wage	1219(537–1899)	115(96–135)	156(116–196)
Medical costs		79.3(12.7–146)	20.0(11.7–28.2)	22.2(15.0–30.4)
Non-medical costs		2239(1742–2737)	198(162–234)	273(199–347)
Indirect costs	Human Capital Approach	1219(538–1901)	116.5(97–136)	157(117–197)
Dissaving/Coping Costs		183(19.0–348)	62.1(48.8–75.5)	66.6(50.3–83.0)
Total		3722(3071–4374)	396(337–456)	519(407–632)

Table 4 shows the coping mechanisms (dissaving) that the TB patients adopt to defray the TB costs, and also shows the social consequences they encounter because of TB. In the survey, up to 571(48.5%) patients used at least one of the 3 dissaving strategies (took a loan, sold assets or used savings) ranging from 536 (47.2%) for DS-TB patients to 35 (81.2%) for MDR-TB. Regarding social consequences, 585 (49.7%) experienced food insecurity, 477 (40.5%) lost a job, 140 (11.8%) had a child interrupt schooling and 633 (53.7%) were socially excluded due to TB and 94 (8%) had divorce or separation from a spouse. The social consequences were worse for the patients in the poorest income quintile and MDR-TB patients. Up to 43.9% of survey households had received a form of social protection after a TB diagnosis was made, with the proportion bigger for MDR-TB patients (56.4%) than for the DS-TB patients (1.8%).

**Table 4:** Dissaving mechanisms and social consequences for sample participants

	Expenditure Quintiles^a^						Treatment Group	
	Poorest	Less Poor	Average	Less Wealthy	Wealthiest	Overall	DS	MDR
	(*N* = 219)	(*N* = 229)	(*N* = 218)	(*N* = 274)	(*N* = 215)	(*N* = 1178)	(*N* = 1134)	(*N* = 44)
**Dissaving Strategies**								
Loan	22.8%	29.1%	27.6%	27%	25.6%	26.3%	25.9%	35.5%
Use of savings	6%	8.6%	12%	14.1%	14.9%	11.2%	10%	39.9%
Sale of assets	29.4%	27.6%	30.1%	25.1%	22%	26.5%	25.4%	54.4%
**Any of the three above**	**45.5%**	**48.6%**	**54.3%**	**48.2%**	**47.6%**	**48.5%**	**47.2%**	**81.2%**
Food insecurity	60.9%	48.6%	49.7%	50%	43.2%	49.7%	49.3%	59.7%
Divorce/separated from spouse/partner	8.7%	6.6%	10.5%	5.3%	9.2%	8%	7.8%	10.6%
Loss of Job	45.1%	44.3%	40.2%	34%	40.9%	40.5%	39.9%	56%
Child interrupted schooling	8.7%	10.7%	11%	11.6%	15.4%	11.8%	11.9%	11.5%
Social exclusion	60%	55.3%	51.4%	53.3%	50.8%	53.7%	54%	46.1%
Any days of work lost	16%	9.2%	4%	2.4%	5.7%	7.2%	4.4%	2.6%
**Household received social protection after TB diagnosis**	3.2%	2.9%	5.1%	4.2%	3.5%	3.9%	1.8%	56.4%

^a^12 people excluded due to zero consumption data

Table 5 presents the proportion of households experiencing catastrophic costs for different households. At a 20% threshold, 614 (53.1%) participants experienced catastrophic costs. The proportion experiencing catastrophic costs increased with lower thresholds at 15 and 10% i.e., 62.4 and 75.2% respectively. The proportion of respondents experiencing catastrophic costs decreased with increased thresholds; 25 and 30% i.e. 45.2 and 38.9% respectively. Regarding direct costs (direct medical and direct non-medical), 33.1% (383) of the respondents spent up to 20% of their annual household income and the same trend as for catastrophic costs was followed with changing thresholds.

**Table 5:** Households facing catastrophic costs

	Expenditure quintiles ^a^					
	Poorest	Less Poor	Average	Less Wealthy	Wealthiest	Overall
	(***N*** = 219)	(***N*** = 229)	(***N*** = 218)	(***N*** = 274)	(***N*** = 215)	(***N*** = 1155)
**Households experiencing total (direct and indirect) costs above (%) - Human capital Approach**						
10%	178 (81.4%)	162 (70.8%)	168 (77.2%)	214 (78.2%)	145 (67.5%)	868 (75.2%)
15%	157 (71.7%)	133 (58.4%)	141 (64.8%)	171 (62.4%)	118 (54.9%)	721 (62.4%)
20%	143 (65.4%)	106 (46.6%)	112 (51.3%)	152 (55.5%)	100 (46.4%)	613 (53.1%)
25%	119 (54.2%)	90 (39.4%)	98 (44.8%)	130 (47.4%)	86 (39.8%)	522 (45.2%)
30%	103 (47%)	76 (33.2%)	84 (38.7%)	117 (42.8%)	68 (31.7%)	449 (38.9%)
**Number of households experiencing direct medical and non-medical costs above (%) annual household expenditure**						
10%	123 (56.1%)	106 (46.2%)	115 (52.9%)	140 (51.2%)	83 (38.6%)	567 (49.1%)
15%	107 (48.9%)	86 (37.5%)	95 (43.4%)	117 (42.6%)	61 (28.5%)	465 (40.3%)
20%	89 (40.7%)	69 (30.4%)	81 (37.2%)	95 (34.8%)	48 (22.2%)	383 (33.1%)
25%	82 (37.3%)	53 (23%)	72 (32.8%)	79 (28.9%)	40 (18.6%)	325 (28.1%)
30%	74 (33.9%)	46 (20.2%)	64 (29.4%)	67 (24.4%)	29 (13.3%)	280 (24.2%)
**Number of households experiencing direct medical costs above (%) annual household expenditure**						
10%	18 (8%)	22 (9.6%)	14 (6.4%)	11 (4%)	7 (3.1%)	71 (6.1%)
15%	11 (5%)	12 (5.4%)	10 (4.3%)	9 (3.3%)	3 (1.4%)	45 (3.9%)
20%	9 (3.8%)	12 (5.4%)	6 (2.5%)	7 (2.6%)	1 (0.5%)	35 (3%)
25%	8 (3.4%)	11 (4.8%)	5 (2.3%)	6 (2.2%)	1 (0.5%)	30 (2.7%)
30%	8 (3.4%)	11 (4.8%)	5 (2.3%)	5 (1.7%)	1 (0.5%)	29 (2.5%)

^a^12 people excluded due to zero consumption data

In terms of direct medical costs, 3% (35) of the households used up to 20% of their annual income for these costs. A similar trend of proportions was followed with adjusted thresholds as for catastrophic costs (i.e., proportions increasing/decreasing) with decreasing/increasing thresholds.

Table 6 illustrates the risk factors for experiencing catastrophic costs. At both bivariate and multivariate analysis, participants belonging to the poorest expenditure quintile had higher odds of experiencing catastrophic costs i.e. bivariate analysis: OR (IQR): 23.5 (12.9–42.7) and multivariate analysis: 24 (13.2–43.8). HIV, age and gender were not associated with higher odds of experiencing catastrophic costs.

**Table 6:** Odds ratios of experiencing catastrophic costs

	Univariate OR (95%CI)	Multivariate OR (95%CI)
**Age**		
0–14	Reference	Reference
15–24	0.6 (0.3–1.3)	0.4 (0.2–0.9)
25–34	0.7 (0.3–1.4)	0.5 (0.2–1)
35–44	0.7 (0.3–1.4)	0.5 (0.2–1)
45–54	0.6 (0.3–1.3)	0.4 (0.2–1)
55–64	0.9 (0.4–1.9)	0.5 (0.2–1.1)
65+	1.2 (0.5–2.8)	0.7 (0.3–1.8)
**Sex**		
Male	1 (0.8–1.4)	1 (0.7–1.3)
Female	Reference	Reference
**Long delay (> 4 weeks before diagnosis)**	1.3 (0.9–2)	1.1 (0.7–1.8)
**HIV Status**		
Positive	1 (0.7–1.4)	1 (0.7–1.3)
Negative	Reference	Reference
**Expenditure Quintile**		
Poorest	23.5 (12.9–42.7)	24 (13.2–43.8)
Less Poor	6.1 (3.9–9.6)	6.2 (4–9.8)
Average	3.9 (2.7–5.8)	4 (2.7–5.9)
Less Wealthy	2.3 (1.6–3.5)	2.3 (1.5–3.4)
Wealthiest (Reference)	Reference	Reference

**Fig. 2:**
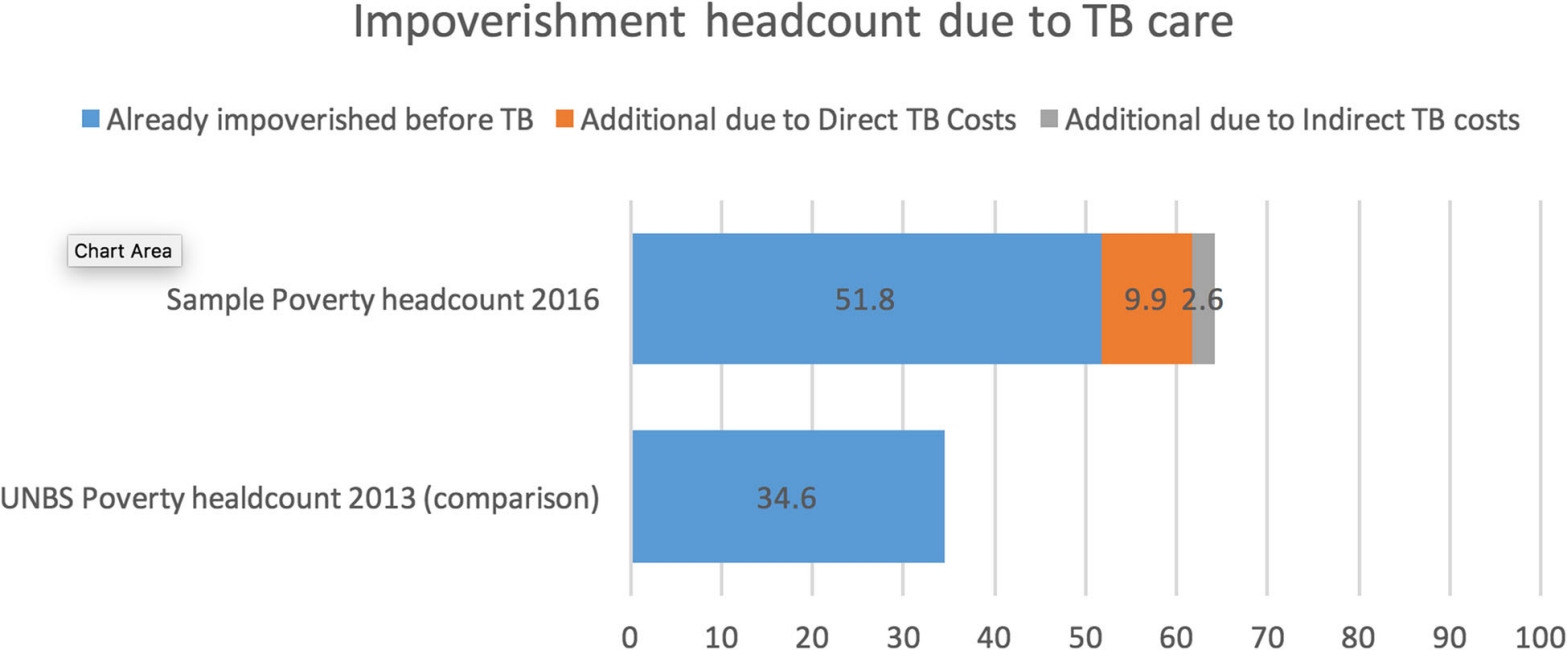
Impoverishment headcount due to TB care

Figure 2 shows the impoverishment due to TB care. Even before TB, 51.8% of the respondents were already below the poverty level. Direct costs pushed an additional 9.9% of the TB patients below the poverty level while the indirect costs pushed an additional 2.6% below the poverty level.

## Discussion

This national TB cost survey established that up to 53% of Ugandan TB affected households incur TB-related costs that are higher than 20% of their annual household expenditures, despite the free TB care policy. The survey also identified the main cost drivers as non-medical expenditure such as travel, nutritional supplements and food.

The proportion of 53% of TB affected households experiencing catastrophic costs is lower than was found in similar studies done in Vietnam, Ghana and Myanmar [7, 12, 13] but higher than was found in Kenya and Indonesia [14, 15]. This difference could be explained by the differences in the geographic, health system and economic profiles of the countries.

TB patients incur direct medical, direct non-medical and indirect costs while they seek care. The study found direct non-medical costs to be the biggest drivers of catastrophic costs, with most of the costs incurred on nutritional supplements, travel and food. This is consistent with findings from similar surveys conducted elsewhere [7, 14, 16]. Data from previous studies have highlighted the contribution of food and transportation to the nearest TB care service on indirect costs; putting the figures at 50 and 37% respectively [17]. A study done in Philippines found out that paying attention to the nutrition costs could reduce the catastrophic costs by 5% [18]. In Uganda, MDR-TB patients receive enablers in form of food and transport vouchers [19]. This survey however shows that despite this, these patients still incur high costs on nutrition and food. Potential solutions could include increasing nutritional and transport support for MDR-TB patients and possibly introducing similar support in the DS-TB patients.

The study found out DS-TB patients spent US$396 for the entire TB episode while DR-TB patients spent up to US$ 3722. Previous work done in Uganda on costs of TB treatment analyzed from health services, patients and community volunteers’ perspective showed the amount needed to successfully treat a new smear-positive TB patient was US$ 911.0 and US$ 391.0 using the hospital-based approach and community-based care approach respectively [20]. The costs incurred by MDR-TB patients in previous surveys have been found to be higher than for DS-TB patients. In Ghana, costs per DS-TB episode were US$429.6 while it was US$659.0 for MDR-TB patients [12]. The amount spent on TB treatment is high in a setting like Uganda where the minimum monthly wage is US$ 36 [21], and 21.4% of the population are below the poverty level [22]. This survey established that even before a TB diagnosis is made, 52% of the TB patients were already below the poverty level, with an additional 12.5% pushed below the poverty level while in TB care. These costs represent a large economic burden to the Ugandan TB affected households, who are financially compromised in the first place.

TB patients adopt several coping measures in a bid to cushion against the TB-related costs. Close to half (48.5%) of the patients had adopted at least one coping mechanism. TB patient cost studies done elsewhere found borrowing money and taking loans were the widely used coping strategies for TB patients [5, 23]. The survey revealed respondents in the lowest income quintiles (poorest, less poor and average) were more likely to take up loans and sell assets as opposed to using up their own savings. This is hardly surprising as this group of patients do not normally have a stable income source compared to individuals in the high-income quintiles and thus hardly have any savings to draw upon.

TB patients encounter several social consequences while in care. In this survey patients experience encountered food insecurity (49.7%), job loss (40.5%), interruption in schooling for children (11.8%) and social exclusion (53.7%). The proportion experiencing these consequences was higher than was found in similar surveys [14, 16], and this could be due to differences in the health care systems, sample sizes and economic profiles of the countries.

In this survey, patients/households belonging in the poorest expenditure quintile had higher odds of experiencing catastrophic costs. TB has often been known as a disease of the poor since the burden follows a strong socio-economic gradient, and also poor communities have been known to have high incidences [23, 24]. TB catastrophic costs are thus disproportionately experienced by individuals who are already at a higher risk of TB. Despite the high proportion of HIV/TB co-infected patients in the survey, HIV didn’t increase the odds of experiencing catastrophic costs. This possibly could be due to the implementation of the one stop shop model for TB/HIV services where TB and HIV services are offered to the clients at the same time and location.

The survey results provide a baseline upon which future catastrophic costs measurements could be compared and progress towards the high-level End TB Strategy target assessed. The survey results are disaggregated by TB resistance status (i.e., DR TB and MDR TB). However, the costs for the MDR TB patients need to be appreciated in context of the low number sampled. For example, the results showed costs incurred by the MDR TB patients for a TB episode are 10 times higher than for DS TB patients. It’s possible there is an over estimation for the MDR TB costs owing to the small number of MDR TB patients included in the survey. Despite this, we believe the costs would still be higher even with bigger numbers as has been seen in other studies that have sampled more MDR TB patients [14, 16, 25].

Based on the survey findings, we recommend a policy shift in order to be able to protect the TB patients against catastrophic costs. This could include operationalization of the national health/social insurance, strengthening and enforcement of legislation related to social protection and intersectoral collaborations as the effects span several sectors.

### Limitations

The survey included a few MDR-TB patients. Subsequent surveys should purposely involve more MDR-TB patients in the sample. Patients also were asked costs previously incurred which might have led to a recall bias. Recall bias mainly affects cost estimates for the pre-treatment period and the approach to only interview persons in intensive phase about diagnostics costs was intended to minimize this type of bias. Also, most of the costs were estimated as the study was cross-sectional in nature. The survey also did not include costs after treatment as some of the direct and indirect costs of TB for the patients and the household can extend beyond the treatment period.

## Conclusion

In conclusion, this survey established that over a half of TB affected households in Uganda face catastrophic TB care expenditure, with the major cost drivers being nutritional supplements, travel, and food. This expenditure results in adverse coping behaviors such as selling assets, taking loans and using savings at high rates among the patients.

## Data Availability

The datasets used and/or analysed during the current study are available from the corresponding author on reasonable request.

## Abbreviations

TB: Tuberculosis
HIV: Human Immunodeficiency Virus
MDR: Multi drug resistant
DTU: Diagnostic and Treatment Unit
USD: United States Dollars
WHO: World Health Organization
DS-TB: Drug Sensitive Tuberculosis
UGX: Uganda Shillings
DOT: Directly Observed Therapy
